# Rising Methicillin-Resistant *Staphylococcus aureus* Infections in Ear, Nose, and Throat Diseases

**DOI:** 10.1155/2014/253945

**Published:** 2014-11-06

**Authors:** Sangeetha Thirumazhisi Sachithanandam

**Affiliations:** Department of ENT, Madha Medical College and Research Institute, Kovoor, Chennai, Tamil Nadu 600122, India

## Abstract

The increasing incidence of methicillin-resistant *Staphylococcus aureus* infections (MRSA) in ENT diseases is becoming a big clinical concern. Here two patients are described who developed MRSA infections presented with unusual post-FESS epistaxis and postmastoidectomy perichondrial abscess and failed treatment with broad spectrum intravenous antibiotics. Following treatment with oral linezolid combined with local mupirocin dressing both patients fully recovered.

## 1. Introduction

Methicillin-resistant* Staphylococcus aureus* (MRSA) has become a serious problem in various diseases such as pneumonia, osteomyelitis, infective endocarditis, and skin and soft tissue infections, including sinonasal and ear infections. Widespread use of broadspectrum antibiotics and previous nasal surgeries contribute much to the emergence of MRSA causing ear and sinonasal infections. Oral linezolid combined with local mupirocin therapy is found to be safe and well tolerated, reduces hospital stay, and is cost effective compared to other antibiotics.

## 2. Case Number 1

A 19-year-old adolescent male with a history of chronic tonsillitis, deviated nasal septum with purulent left maxillary sinusitis, underwent tonsillectomy in January 2014 and had a hospital stay in general ward for 5 days inclusive of preoperative and postoperative period, which was uneventful. Six weeks later, he underwent septoplasty with functional endoscopic sinus surgery. Frank pus was drained out from the left maxillary sinus and normal saline wash was given to clear the pus completely. On 2nd postoperative day patient developed unusual nasal bleeding on merocel pack removal. He was conservatively managed with regular nasoendoscopic suctioning but he continued to have epistaxis. Nasal endoscopy revealed severe inflammation with edematous nasal mucosa and generalized oozing from the lateral wall of nose and septal flaps. He was empirically started with intravenous Augmentin 1.2 gm twice daily. Nasal swab cultures were positive for MRSA, which was sensitive to vancomycin, rifampicin, linezolid, daptomycin, and tetracycline but resistant to penicillin, ciprofloxacin, clindamycin, levofloxacin, erythromycin, and oxacillin. He was started on oral linezolid 600 mg twice daily for 10 days with local mupirocin ointment application. He showed improvement within 48 hours and there was no further oozing. His nasal mucosa healed well with clear sinuses in sequential endoscopies during the 6 weeks of follow-up.

## 3. Case Number 2

An 18-year-old adolescent male with left ear chronic suppurative otitis media with atticoantral cholesteatoma admitted and underwent left modified radical mastoidectomy with type 3 tympanoplasty in July 2013. He was discharged on 7th postoperative day. He had a hospital stay for 9 days inclusive of preoperative and postoperative period which was uneventful. After 2 weeks of surgery he complained of severe pain, swelling of left auricle. On examination, the left auricle was tender, tense and edematous. Operated mastoid cavity had remnant gelfoam in situ. He was readmitted for postoperative perichondritis left ear and empirically started on intravenous Augmentin 1.2 gm twice daily but it turned into perichondrial abscess within 48 hours, showing no improvement. Abscess was drained out. Swab cultures were positive for MRSA. Oral linezolid 600 mg twice daily combined with local mupirocin ointment dressing was done for 10 days. There was significant improvement with no further pus collection. Perichondritis resolved completely and the patient was discharged.

## 4. Discussion

With the increasing incidence of MRSA in ear, throat, and sinonasal infections not much is known about the best way to manage it. The probable risk factor was previous nasal surgeries in adults and previous increased frequency of antibiotic usage in children [1, 2]. Both patients with MRSA infections described above were operated for chronic maxillary sinusitis and unsafe chronic suppurative otitis media. Both had the history of chronic antibiotic usage for their infections before getting operated. Studies have documented the recovery of MRSA from the core and surface of tonsils removed because of recurrent group A beta hemolytic streptococcus tonsillitis [3]. Throat carriage of MRSA in hospital staffs with pharyngeal tonsillitis was reported in earlier studies [4]. It may serve as a potential source for the spread of these organisms to other body sites as well. The above described patient with sinonasal MRSA infection underwent tonsillectomy 6 weeks before the surgery which may serve as a source of infection to sinuses. Also MRSA infections appeared to be more common in chronic otitis media than on acute infections. The prevalence of MRSA in infections in discharging ears represents an increasing problem [5]. The frequency of MRSA was found to be significantly higher in adults with otitis media than in children [6].

The vast majority of MRSA infections are acquired in hospitals more with long term stay. The main reservoir of MRSA in hospitals is patients colonized or infected with MRSA. Like other strains of* S. aureus*, the body site most commonly colonized with MRSA is the anterior nares. About 40% to 60% of hospitalized patients colonized with MRSA develop an overt infection. These infections are associated with prolonged hospital stays [7]. Both patients described above had a hospital stay of minimum 9 days in general ward. Nosocomial transmission may also be a probable source of infection.

Intravenous vancomycin and daptomycin are considered to be the first line antibiotic choices for MRSA bacteraemia [8, 9]. The emergence of vancomycin resistant MRSA proves the necessity for new generation antibiotics like linezolid in the treatment of MRSA infections [10].

Linezolid, a member of oxazolidinone class of drugs, is a synthetic antibiotic active against most gram positive bacteria that are resistant to several other antibiotics. As a protein synthesis inhibitor the exact mechanism of action of linezolid is unique in the fact that it blocks the initiation step unlike other protein synthesis inhibitors which inhibit elongation.

One of the advantages of linezolid is its high bioavailability (close to 100%). When given by mouth the entire dose reaches the blood stream. Linezolid has low plasma protein binding (approximately 31%); apparent volume of distribution at steady state is around 40–50 litres. Linezolid demonstrates adequate penetration into the tissues with sustained concentrations above the minimum inhibitory concentrations for majority of the dosing interval [11]. Linezolid was proved to consistently achieve microbiological eradication in MRSA patients [12].

Oral linezolid therapy is safe and well tolerated, reduces hospital stay, and is cost effective compared to vancomycin and other parenteral antibiotics for MRSA infections [13, 14]. Pharmacoeconomic studies have demonstrated an overall reduction in total direct costs to the payer in favour of linezolid over its comparators [15, 16]. When administered for shorter periods linezolid is a safe drug. It can be used in patients of all ages, people with liver disease or poor kidney function.

Patients with serious underlying diseases often remain colonized in the anterior nares for a prolonged time, often several years.

Currently, the agent of choice for eradicating MRSA nasal carriage is mupirocin ointment. Application of a small amount of ointment in the anterior nares 2 to 3 times a day for 5 days is often effective [7, 17]. Local mupirocin application proved to be useful in the management of MRSA exacerbation of chronic rhinosinusitis and perichondrial abscess. Long term intranasal mupirocin treatment in MRSA carrier patients with long hospital stay was proved to decrease acquired carriage and MRSA infections [18–21].

## 5. Conclusion

Oral linezolid with local mupirocin is found to be a good alternative to its intravenous comparators for MRSA complicated sinonasal and ear infections.

## References

[1] Huang W.-H., Hung P.-K. (2006). Methicillin-resistant Staphylococcus aureus infections in acute rhinosinusitis. *Laryngoscope*.

[2] Manarey C. R. A., Anand V. K., Huang C. (2004). Incidence of methicillin-resistant *Staphylococcus aureus* causing chronic rhinosinusitis. *The Laryngoscope*.

[3] Brook I., Foote P. A. (2006). Isolation of methicillin resistant *Staphylococcus aureus* from the surface and core of tonsils in children. *International Journal of Pediatric Otorhinolaryngology*.

[4] Van Der Vorm E. R., Groenendijk E. H. (2003). Two hospital staff with throat carriership of meticillin-resistant *Staphylococcus aureus*, which had to be treated with systemic antibiotics. *Nederlands Tijdschrift voor Geneeskunde*.

[5] Hwang J.-H., Tsai H.-Y., Liu T.-C. (2002). Community-acquired methicillin-resistant *Staphylococcus aureus* infections in discharging ears. *Acta Oto-Laryngologica*.

[6] Lee J. S., Kim M. G., Hong S. M., Na S. Y., Byun J. Y., Park M. S., Yeo S. G. (2014). Changing patterns of bacterial strains in adults and children with otitis media in Korean tertiary care centers. *Clinical and Experimental Otorhinolaryngology*.

[7] (1998). *Clinical Updates in Infectious Diseases*.

[8] Holland T. L., Arnold C., Fowler V. G. (2014). Clinical management of *Staphylococcus aureus* bacteremia. *The Journal of the American Medical Association*.

[9] Rehm S. J., Boucher H., Levine D., Campion M., Eisenstein B. I., Vigliani G. A., Corey G. R., Abrutyn E. (2008). Daptomycin versus vancomycin plus gentamicin for treatment of bacteraemia and endocarditis due to *Staphylococcus aureus*: subset analysis of patients infected with methicillin-resistant isolates. *Journal of Antimicrobial Chemotherapy*.

[10] Pangercić A., Bukovski-Simonoski S., Barsić B. (2010). Lipopeptides and oxazolidinones—novel antibiotics in MRSA infection treatment. *Liječnički Vjesnik*.

[11] Stein G. E., Wells E. M. (2010). The importance of tissue penetration in achieving successful antimicrobial treatment of nosocomial pneumonia and complicated skin and soft-tissue infections caused by methicillin-resistant Staphylococcus aureus: vancomycin and linezolid. *Current Medical Research and Opinion*.

[12] Bounthavong M., Hsu D. I. (2010). Efficacy and safety of linezolid in methicillin-resistant *Staphylococcus aureus* (MRSA) complicated skin and soft tissue infection (cSSTI): a meta-analysis. *Current Medical Research and Opinion*.

[13] Stevens D. L., Herr D., Lampiris H., Hunt J. L., Batts D. H., Hafkin B. (2002). Linezolid versus vancomycin for the treatment of methicillin-resistant Staphylococcus aureus infections. *Clinical Infectious Diseases*.

[14] McCollum M., Sorensen S. V., Liu L. Z. (2007). A comparison of costs and hospital length of stay associated with intravenous/oral linezolid or intravenous vancomycin treatment of complicated skin and soft-tissue infections caused by suspected or confirmed methicillin-resistant *Staphylococcus aureus* in elderly US patients. *Clinical Therapeutics*.

[15] Bounthavong M., Hsu D. I. (2012). Cost-effectiveness of linezolid in methicillin-resistant *Staphylococcus aureus* skin and skin structure infections. *Expert Review of Pharmacoeconomics & Outcomes Research*.

[16] Grau S., Rubio-Terrés C. (2008). Pharmacoeconomics of linezolid. *Expert Opinion on Pharmacotherapy*.

[17] Solares C. A., Batra P. S., Hall G. S., Citardi M. J. (2006). Treatment of chronic rhinosinusitis exacerbations due to methicillin-resistant *Staphylococcus aureus* with mupirocin irrigations. *The American Journal of Otolaryngology—Head and Neck Medicine and Surgery*.

[18] Sloot N., Siebert J., Höffler U. (1999). Eradication of MRSA from carriers by means of whole-body washing with an antiseptic in combination with mupirocin nasal ointment. *Zentralblatt fur Hygiene und Umweltmedizin*.

[19] Rohr U., Mueller C., Wilhelm M., Muhr G., Gatermann S. (2003). Methicillin-resistant Staphylococcus aureus whole-body decolonization among hospitalized patients with variable site colonization by using mupirocin in combination with octenidine dihydrochloride. *Journal of Hospital Infection*.

[20] Dupeyron C., Campillo B., Richardet J. P., Soussy C. J. (2006). Long-term efficacy of mupirocin in the prevention of infections with meticillin-resistant *Staphylococcus aureus* in a gastroenterology unit. *Journal of Hospital Infection*.

[21] Ridenour G., Lampen R., Federspiel J., Kritchevsky S., Wong E., Climo M. (2007). Selective use of intranasal mupirocin and chlorhexidine bathing and the incidence of methicillin-resistant *Staphylococcus aureus* colonization and infection among intensive care unit patients. *Infection Control and Hospital Epidemiology*.

